# Book Reviews

**Published:** 1890-04

**Authors:** 


### Book Reviews.

A Treatise on Fractures. By Prof. Armand Despees. Translated by E. P. Hurd, M. D. Published in the Physician's Leisure Library. A little volume of 112 pages, with a number of illustrations.

This is a very brightly written, readible book, giving as it does the result of the author's ripe experience in the clinical treatment of fractures. As he says in the beginning, it is not designed to be a complete treatise on fractures, but only to give those methods of treatment that have given him the best results in his own practice.

The writer has treated his subject with much independence and originality. In some parts with too much of both, especially in regard to his methods of treatment, which do not come up to the approved methods now in use in this country, either in comfort to the patient or results in the treatment of the fractures.

Saunders' Question Compends. Essentials of Gynæcology. By Edwin B. Cragin, M. D.

This little book is fully up to the standard of this series, and will no doubt be reviewed with the same pleasure that the preceding volumes have.

Syllabus of Obstetrical Lectures. By Richard C. Norris, M. D. W. B. Saunders, Philadelphia.

This little work is surely a great aid to the student, and fills the place it was intended for most admirably.

### DISEASES OF WOMEN AND ABDOMINAL SURGERY.

BY LAWSON TAIT, F. R. C. S., ETC. NOL. I. PHILADELPHIA. LEA BROS. & CO. 1889.

In the treatise of which this is the first volume Tait has presented to the world the results of his individual works in gynæcology. That there is much originality in the work goes without saying, for no man respects less than Tait the traditions of the elders. It is this feature which gives to the book its value, since the views presented are the outcome of personal observation, and are never taken at second hand. By reason of this fact we can readily overlook the rancor, the intolerance, the belligerency which characterizes this, as well as all of Tait's writings, and separating the large amount of wheat from the small amount of chaff, accord to the work the praise which it deserves. The profession is indebted to Tait more than to any other man for the impetus which has been given to abdominal surgery in the last decade, and the same bold spirit which has made him *facile princeps* in laparotomy will be found to have characterized his methods in all departments of gynæcology. The work is therefore a valuable one, as offering new modes of treatment which may be found applicable to cases where the recognized treatment has failed, and there is no hazard in predicting that many of the new methods herein proposed will meet with favor at the hands of the profession. The author concludes the preface of the work in the following well-spoken words:

"Nor can I imagine it possible that any modern gynæcologist, whether he accepts my views or not, could conscientiously say that my work has not been extensive enough to entitle me to express my convictions concerning it." V. O. H.

---

OLD ALE.—Some beer was recently discovered, walled up in the cellars of a brewery at Burton-on-Trent, which had been brewed in the year 1798. It was found to be in good condition, resembling sherry more than it did a malt liquor.

---

The Tennessee State Medical Society will hold its fifty-seventh annual meeting April 8th, 9th and 10th in Chattanooga. The Society was organized in 1831, and this would have been the sixtieth meeting had not the war put an interruption to the annual reunions for a time.

---

It is said that the practice of drinking cologne is becoming very common in Europe and in this country, and as an indication of this that the sale of the perfume has increased greatly of late years. Women are more addicted to the habit than men, and a writer in the *Quarterly Journal of Inebriety* says that the presence of obscure and complex nervous disorders in a woman who uses cologne externally should always sugges the possibility of its internal use.—*Gaillard Medical Journal.*

